# Pediatricians' weight assessment and obesity management practices

**DOI:** 10.1186/1471-2431-9-19

**Published:** 2009-03-05

**Authors:** Jeannie S Huang, Michael Donohue, Golnaz Golnari, Susan Fernandez, Edward Walker-Gallego, Kate Galvan, Christina Briones, Jennifer Tamai, Karen Becerra

**Affiliations:** 1 Department of Pediatrics, University of California, San Diego, CA 92103, USA; 2 Rady Children's Hospital, San Diego, CA, USA; 3 Division of Biostatistics and Bioinformatics, University of California, San Diego, La Jolla, CA, USA

## Abstract

**Background:**

Clinician adherence to obesity screening guidelines from United States health agencies remains suboptimal. This study explored how personal and career demographics influence pediatricians' weight assessment and management practices.

**Methods:**

A web-based survey was distributed to U.S. pediatricians. Respondents were asked to identify the weight status of photographed children and about their weight assessment and management practices. Associations between career and personal demographic variables and pediatricians' weight perceptions, weight assessment and management practices were evaluated using univariate and multivariate modeling.

**Results:**

3,633 pediatric medical providers correctly identified the weight status of children at a median rate of 58%. The majority of pediatric clinicians were white, female, and of normal weight status with more than 10 years clinical experience. Experienced pediatric medical providers were less likely than younger colleagues to correctly identify the weight status of pictured children and were also less likely to know and use BMI criteria for assessing weight status. General pediatricians were more likely than subspecialty practitioners to provide diverse interventions for weight management. Non-white and Hispanic general practitioners were more likely than counterparts to consider cultural approaches to weight management.

**Conclusion:**

Pediatricians' perceptions of children's weight and their weight assessment and management practices are influenced by career and personal characteristics. Objective criteria and clinical guidelines should be uniformly applied by pediatricians to screen for and manage pediatric obesity.

## Background

Overweight in children is a public health concern in the United States (U.S.) with increasing prevalence rates among children of all ages and cultural backgrounds over the past two decades [1]. Globally, it is estimated that 22 million children under the age of 5 are overweight [2]. The associations between overweight in children and significant health morbidities [3-7] have resulted in national health campaigns urging medical providers' involvement in the detection and treatment of this growing epidemic.

Although the Department of Health and Human Services [8], the American Academy of Pediatrics [9] and the American Medical Association [10] strongly advocate the use of objective criteria for obesity detection among children, clinician adoption of these guidelines remains suboptimal [11-18] and BMI documentation rates vary widely, from 0.5% [14] to 52% [18]. We sought to determine whether personal and career demographics influenced U.S. pediatricians' weight assessment and management practices. Our a priori hypothesis was that U.S. pediatricians' weight assessment and management practices are influenced by personal and career demographics.

## Methods

### Participants and Setting

The study was approved by the University of California, San Diego Human Research Protections Program (UCSD HRPP). Potential participants were identified via membership in a non-profit U.S. pediatric medical association consisting of general pediatricians, pediatric subspecialists, and ancillary pediatric medical care providers. Members were eligible if they had a valid email address and were directly involved in the healthcare of children.

### Study Performance

Potential participants were invited via e-mail to join a closed, password-protected, web-based survey. Unique passwords were assigned to prevent duplicate survey entries from a single participant. A nominal gift card was offered for participation. Personal and career demographic data were collected. Participant body mass index (BMI) was calculated from self-reported height and weight. Participants answered questions regarding their weight assessment and weight management practices. Specific questions and response options are presented in Table 1. Participants were also shown photographs of children (described in photograph questionnaire) and asked to determine the weight status of pictured children (response options: underweight, normal weight, overweight, or obese).

**Table 1:** Weight assessment and weight management practices of surveyed pediatricians (N = 3,633).

Weight status assessment method (%)	10 General appearance
	3 Weight alone
	21 Weight for height
	64 Body mass index
	2 Professional experience
Knowledge of NCHS criterion for overweight (%)	41

Knowledge of NCHS criterion for obese (%)	43

Frequency of calculating body mass index (%)	11 At all visits
	35 Only at well-child visits
	34 Only when concerned
	11 Never
	9 Only after a certain age

Frequency of weight concern discussion with patients (%)	18 At every visit
	17 Only at well-child visits
	62 Only when concerned
	2 Never
	1 Only after a certain age

Interventions provided for weight management to patients (%)	82 Nutrition
	54 Physical Activity
	17 Social Work
	62 Counseling
	20 Support Groups

Consideration for Culture in Weight Management	37 Yes
Approach	63 No

### Photograph questionnaire

The questionnaire consisted of 12 photographs of children in four age categories (3 photographs per category: infant, toddler (1–3 years), 4–6 years, and 10–12 years). Children were photographed in both the anteroposterior and lateral positions in their undergarments. Weight status for photographed children ≥ 2 years of age were defined according NCHS BMI-for-age and sex percentile definitions of underweight (≤ 5%), normal weight (5%< × < 85%), overweight (85% ≤ × < 95%) and obese (≤ 95%) [19]. Children <2 years of age were similarly assigned weight status based on sex-specific weight-for-height percentiles and the following definitions: underweight (≤ 5%), normal weight (5%< × < 85%), overweight (85% ≤ × <95%) and obese (≤ 95%).

### Response Coding

Racial response categories included: white, black, Hispanic, Asian, or other; for statistical analyses, these groups were collapsed according to white or non-white and Hispanic or non-Hispanic origins. Other variables were also collapsed into dichotomous categories, including: sex (male vs. female); number of family generations in the United States (≥ 2 vs. <2); participant weight status (overweight and obese vs. normal weight and underweight); and career experience (<5 vs. ≥ 5 years). Participant weight category was assigned according to NCHS BMI categorical definitions [20].

Characteristics of photographed children were coded as follows: child sex (male vs. female); child race (white, Hispanic, or non-white non-Hispanic); child age (infant or toddler vs. 4–6 or 10–14 years); and child weight status (overweight or obese vs. normal weight or underweight).

Responses for the photograph survey were coded as correct or incorrect and as underestimation vs. overestimation or correct. Pediatric medical provider ability to correctly identify the weight status of children was represented as the number of photographed children for whom weight status was correctly identified (out of 12). Ability to correctly identify the weight status of overweight or obese unrelated children was represented as the number of photographed overweight or obese children correctly identified (out of 7).

Weight assessment practices were dichotomously coded as follows: whether the survey participant correctly identified *both *NCHS definitions for overweight and obese or not, whether the participant used subjective or objective criteria to assess weight status, whether the participant plotted BMI on a regular or selective basis, and whether the participant discussed weight concerns on a regular or selective basis. Weight management practices were coded as follows: whether the survey participant offered patients the weight management service (i.e. dietary services) or not, and whether the participant considered cultural issues or not in his/her approach to weight management.

### Statistical analyses

Only complete data from pediatricians who confirmed direct participation in the healthcare of children were entered into the analysis (N = 3,633). Demographic data were summarized using descriptive statistics. Univariate analyses of participants' ability to correctly identify children's weight status by selected factors were performed using ANOVA analyses. Multivariate regression analysis was then applied to identify predictors of medical providers' ability to correctly identify children's weight that were independently significant after adjusting for other variables. Only variables with significant associations (defined as p < 0.05) were kept in the final model.

In order to assess the association between photographed children's characteristics and participants' ability to correctly identify the weight status of children (Table 2), the generalized estimating equation (GEE) approach [21] was used with logistic regression owing to correlated outcome data (since each participant rated all of the 12 unrelated children's weight status). For logistic regression analyses, odds ratios (OR's) were calculated to determine the likelihood of the examined outcome.

**Table 2:** Univariate GEE Logistic Regression Analyses of surveyed practitioners' ability to estimate the weight status of photographed unrelated children and Selected Characteristics of the Photographed Children.

**Photographed Child Characteristics**	**OR (95% CI) (correct vs. incorrect)**	**OR (95% CI) (underestimate vs. correct or overestimate)**
**Age (4–6 years or 10–14 years vs. Infant or Toddler)**	8.76 (8.17, 9.39)	0.07 (0.07, 0.08)

**Race (white vs. Non-white, non-Hispanic)**	0.23 (0.22, 0.24)	0.42 (0.39, 0.46)

**Race (Hispanic vs. Non-white, Non-Hispanic)**	0.60 (0.56, 0.64)	0.33 (0.30, 0.35)

**Sex (Female vs. Male)**	1.20 (1.15, 1.26)	1.39 (1.25, 1.55)

**Weight Status (Overweight or obese vs. Normal weight or underweight)**	0.16 (0.15, 0.17)	143 (116, 176)

Results are presented as odds ratio (95% confidence intervals). Odds ratios represent the odds of participants correctly vs. incorrectly identifying the weight of children according to selected variables.

Participants' weight assessment and management practices were analyzed by selected factors using logistic regression analysis. Odds ratios (OR's) were calculated to determine the likelihood of the examined outcome by selected factors.

Statistical analyses were performed on questionnaire responses using JMP 5.0 statistical software (Cary, NC) and the R statistical package version 2.6.1 [22,23]. Significance for all analyses was set at p < 0.05.

## Results

4,945 pediatric medical providers answered the web-based questionnaire, with a participation rate of 94.6%, as defined by the Checklist for Reporting Results of Internet E-surveys [24], and 4,841 completed the questionnaire, with a completion rate of 97.9%. For this analysis, only data from the 3,633 participating pediatricians were analyzed. Demographics, weight assessment and management practices of the 3,633 pediatricians are presented in Tables 1 and 3.

**Table 3:** Demographic data of surveyed pediatricians (N = 3,633).

Sex (M:F, %)	44: 56
BMI (kg/m^2^)	24.5 ± 4.0

Actual Weight status (based on self reported height and weight – %)	40 Overweight/Obese
	58 Normal
	2 Underweight

Race (%)	77 White
	12 Asian
	3 Black
	5 Hispanic
	3 Other

US Geographical Region (%)	15 Midwest
	25 Northeast
	39 South
	21 West

# Family Generations in the United States (%)	21 One
	79 Two or More

Professional Status (%)	76 General
	24 Subspecialist

Years Professional Experience (%)	16 <5 years
	27 5–10 years
	57 > 10 years

*Results expressed as mean ± standard deviation.

### Weight perceptions

Surveyed pediatric medical providers were able to correctly identify weight status in 7 (6, 8) [median (interquartile range)] out of 12 of presented photographs. In univariate analyses, respondents who were female (p = 0.04) and had less professional experience (<5 years, p < 0.01) were more likely than category counterparts to correctly assess children's weight status based on appearance alone. In multivariate analysis, only fewer years of professional experience remained significantly associated with ability to correctly assess children's weight based on appearance alone (p = 0.015).

Surveyed medical providers correctly identified overweight and obesity in 3 (3, 4) out of 7 presented photographs of overweight or obese children. Pediatricians with less professional experience (p = 0.002) were more likely than category counterparts to correctly assess overweight and obesity as compared to category counterparts.

Pediatricians were more likely to correctly (versus incorrectly) assess the weight status of photographed children if the children were non-white, non-Hispanic, female, normal weight or underweight, and 4 years or older (Table 2; column 1). Practitioners were more likely to underestimate (versus correctly estimate or overestimate) the weight status of photographed children if children were female, non-white, non-Hispanic, and infants or toddlers (Table 2, column 2).

### Weight assessment and management practices

The majority of surveyed pediatricians utilized objective methods to assess the weight of children, and BMI was the most common objective method used. However, only 46% physicians calculated BMI routinely (at all visits or at well-child visits) and even fewer (31%) physicians knew the NCHS definitions for both overweight and obesity among children. Weight assessment (Table 4) practices varied according to personal and career demographics. Specifically, female, non-overweight, and general pediatricians with fewer years of career experience (<5 years) were more likely than counterparts to know and use objective criteria to assess weight. General pediatricians were more likely than subspecialists to calculate BMI only at well-child visits, and subspecialists were more likely than general pediatricians to calculate BMI at every visit.

**Table 4:** Univariate Analyses of surveyed practitioners' weight assessment practices and Selected Characteristics of the Practitioners

**Practitioner Characteristic**	**Use of Objective Criteria to Assess Weight**	**Knowledge of NCHS definitions of weight status**	**Calculation of BMI at every visit**	**Calculation of BMI at well-child visits only**
**Sex (Male vs. Female)**	0.58 (0.48, 0.72)	0.76 (0.66, 0.88)	1.01 (0.83, 1.25)	0.78 (0.69, 0.89)

**Race (white vs. Non-white)**	0.83 (0.65, 1.08)	1.03 (0.86, 1.22)	0.76 (0.6, 0.96)	1.07 (0.92, 1.26)

**Ethnicity (Non-Hispanic vs. Hispanic)**	0.52 (0.28, 0.96)	1.11 (0.79, 1.56)	0.74 (0.47, 1.14)	0.88 (0.65, 1.20)

**Weight Status (Overweight or Obese vs. Normal weight or Underweight)**	0.81 (0.66, 1)	0.81 (.70, 0.94)	0.88 (0.71, 1.09)	0.86 (0.75, 0.98)

**Professional Experience (<5 years vs. ≥ 5 years)**	1.73 (1.25, 2.40)	1.65 (1.38, 1.98)	1.27 (0.98, 1.65)	1.39 (1.18, 1.67)

**Subspecialty Status (Generalist vs. Subspecialist)**	3.18 (2.57, 3.92)	1.48 (1.24, 1.76)	0.52 (0.42, 0.65)	3.57 (3, 4.17)

Results are presented as unadjusted odds ratio (95% confidence intervals). Odds ratios represent the odds of participants with selected variables performing/knowing vs. not performing/knowing the recommended guidelines.

In regards to weight management practices, the majority of physicians discussed weight-related issues "only when concerned." However, physicians who reported calculating BMI at well-child visits were more likely to counsel patients regarding weight than those who did not calculate BMI at well-child visits [OR = 1.37 (1.20, 1.57), calculate BMI at well-child visits vs. not]. In the context of weight management, most pediatricians provided nutritional, physical activity, and counseling interventions. Non-white, Hispanic, female and younger general pediatricians were more likely than counterparts to provide cultural approaches to weight management (Table 5). General pediatricians were more likely than subspecialty practitioners to provide diverse interventions with cultural considerations for weight management (Table 5). Weight management practices did not vary according to pediatricians' personal weight status.

**Table 5:** Univariate Analyses of surveyed practitioners' weight management practices and Selected Characteristics of the Practitioners

**Practitioner Characteristic**	**Provision of Nutritional Intervention**	**Provision of Counseling**	**Provision of Support Groups**	**Provision of Social Work Intervention**	**Provision of Physical Activity Intervention**	**Cultural Considerations in Weight Management Approach**
**Sex (Male vs. Female)**	0.90 (0.76, 1.07)	1.13 (0.98, 1.29)	0.89 (0.75, 1.05)	0.89 (0.74, 1.06)	1.33 (1.16, 1.51)	0.84 (0.74, 0.96)

**Race (white vs. Non-white)**	0.80 (0.65, 0.99)	0.78 (0.66, 0.92)	0.93 (0.76, 1.13)	0.77 (0.63, 0.94)	0.68 (0.58, 0.79)	0.63 (0.54, 0.74)

**Ethnicity (Non-Hispanic vs. Hispanic)**	0.88 (0.58, 1.33)	0.83 (0.60, 1.15)	0.69 (0.49, 0.99)	0.92 (0.62, 1.37)	0.90 (0.66, 1.22)	0.67 (0.49, 0.91)

**Professional Experience (<5 years vs. ≥ 5 years)**	1.22 (0.96, 1.56)	0.77 (0.64, 0.92)	0.90 (0.72, 1.14)	1.15 (0.91, 1.45)	1.22 (1.01, 1.45)	1.40 (1.17, 1.67)

**Subspecialty Status (Generalist vs. Subspecialist)**	1.92 (1.61, 2.33)	1.56 (1.35, 1.85)	1.25 (1.02, 1.52)	0.46 (0.38, 0.56)	1.30 (1.11, 1.52)	1.41 (1.20, 1.66)

Results are presented as unadjusted odds ratio (95% confidence intervals). Odds ratios represent the odds of participants categorized according to selected variables offering vs. not offering certain services for weight management.

## Discussion

To our knowledge, we present the largest study evaluating pediatricians' weight perceptions of children and their weight assessment and management practices. Our study also assessed a number of personal and career demographic variables in order to control for potential confounding factors. The implications of our findings are discussed below.

In our U.S. sample, pediatricians were not proficient at discerning children's weight status based on appearance alone, especially among children who were overweight or obese. In comparison to parents who took the same survey, pediatricians as a group demonstrated the exact same accuracy rate at weight status identification as parents [25]. Practitioners also demonstrated weight perception biases according to the characteristics of the children evaluated. Fortunately, only a minority of pediatricians reported using subjective criteria to judge weight status in children.

Use of BMI criteria for weight screening among surveyed pediatricians was suboptimal. Less than half of surveyed pediatricians knew the NCHS definitions for pediatric overweight and obesity, and a minority of physicians reported calculating BMI on a routine basis (either at well-child care visits or at all visits). Our findings validate prior studies documenting low BMI screening among pediatricians [17,26].

Surveyed physicians dispersed weight-related interventions based on level of concern. Other studies also demonstrate discretionary weight-related counseling by physicians [12,17]. Universal screening for pediatric overweight and obesity would increase weight-related counseling by physicians by improving identification of overweight and obesity. In our cohort, physicians who reported regular screening for pediatric obesity at well-child care visits were more likely to counsel on weight than physicians who did not. Recent guidelines also advocate that weight-related issues such as diet and physical activity be discussed regardless of weight status [9].

Our data indicate that practitioner characteristics need to be addressed when developing interventions aimed at improving provider compliance with obesity management recommendations. Both weight assessment and weight management practices varied according to physician characteristics. Our finding that overweight practitioners were less likely to know weight assessment definitions and routinely use BMI to assess weight validates prior data demonstrating reduced obesity recognition by overweight vs. normal weight practitioners [27]. Younger practitioners' familiarity with and use of BMI screening for weight assessment may reflect more recent training and formal review of current weight assessment guidelines. Cultural considerations in weight management by junior, non-white, and Hispanic health practitioners may reflect greater acceptance of and/or personal familiarity with cultural issues. Lastly, increased use of multidisciplinary approaches for weight management by general vs. subspecialty pediatric health professionals may reflect greater physician awareness of weight-related issues resulting from more frequent and intimate contact in the primary care vs. subspecialty setting. Given the chronicity of weight management, interdisciplinary coordination is often required to successfully accomplish health-related goals.

### Limitations

The findings of this study are subject to a number of limitations. Most importantly, study data were retrieved from a small representation of currently practicing providers who interact with children. We nevertheless present data from the largest cohort to date of U.S. pediatric medical practitioners. In addition, we chose to determine weight status from self-reported parameters. However, prior studies have shown that self-reported weight and height are reliable for determining weight status, particularly among physicians [28,29]. Thirdly, weight assessment and management practices were self-reported by surveyed practitioners. However, in the case of response bias, we would expect that surveyed practitioners would over-report practices known to comply with expert recommendations; therefore, actual compliance with expert recommendations may in fact be even more suboptimal than documented in this report. Lastly, lack of provision of weight management resources by providers may have reflected poor availability of resources. Nevertheless, we would have expected that resources would not vary by physician characteristics as was demonstrated in our cohort.

## Conclusion

In conclusion, we demonstrate that U.S. pediatricians' weight assessment and management practices are associated with personal and career demographics. U.S. clinicians who regularly evaluate children should use objective weight screening methods and criteria. Similarly, pediatricians should adopt expert clinical guidelines to minimize personal bias in the management of weight.

## Competing interests

The authors declare that they have no competing interests.

## Authors' contributions

JH conceived of the study, participated in its design, coordination, and data analysis, and helped draft the manuscript. MD helped perform the statistical analysis and helped draft the manuscript. EWG, KG, CB, GG, SF, and JT recruited participants, and helped with data integrity and analysis. KB coordinated study recruitment, carried out the survey performance, and supervised data integrity and analysis. All authors read and approved the final manuscript.

## Pre-publication history

The pre-publication history for this paper can be accessed here:


